# Supplementary figures and images for: Recruitment of the CoREST transcription repressor complexes by Nerve Growth factor IB-like receptor (Nurr1/NR4A2) mediates silencing of HIV in microglial cells
Source: PLoS Pathog. 2022 Jul 7;18(7):e1010110. doi: 10.1371/journal.ppat.1010110 (PMC9295971; doi:10.1371/journal.ppat.1010110)

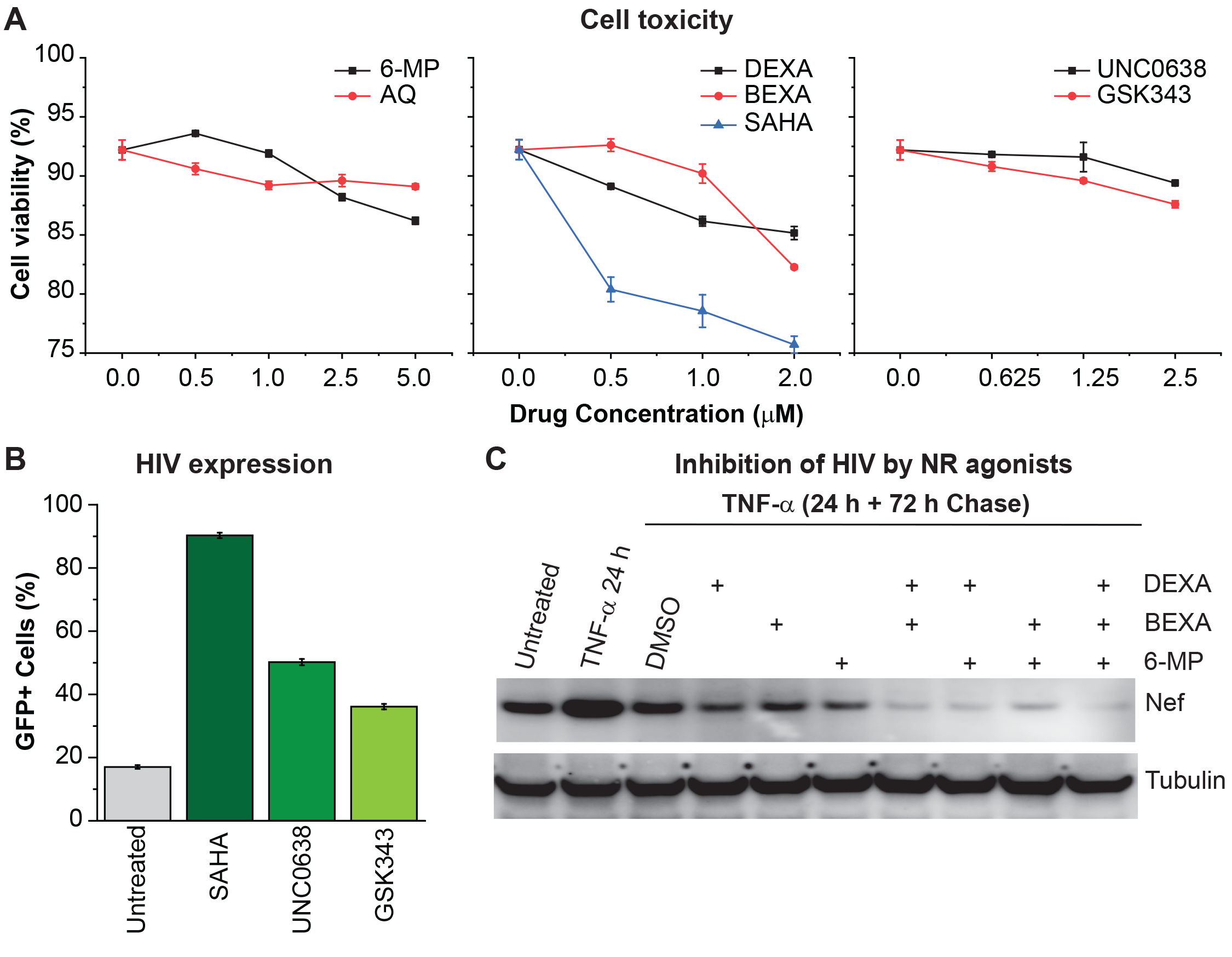

Supplement: S1 Fig — HC69 cells were cultured in the absence or presence of the different chemicals at various concentrations for 48 hr. Cell viability of the differently treated cells was analyzed by using the eBioscience Fixable Viability Dye eFluor 450 kit (Thermo Fisher Scientific, Cat# 65–0863) and measured by flow cytometry. The average number of viable cells from each sample was calculated from triplicate assays of a single experiment. A, Viability of HC69 cells. Left: Cells treated with Nurr1 agonists 6-MP and AQ at 0 to 5 μM for 48 hr. Center: Cells treated with GR agonist DEXA, the RXR agonist BEXA, and the HDAC inhibitor SAHA at 0.5 to 2 μM for 48 hr. Right: Cells treated with G9a and EZH2 inhibitors UNC0638 and GSK343 at 0.625 to 2.5 μM for 48 hr. B, GFP expression in HC69 cells following treatment with SAHA (1 μM), UNC0638 (1.25 μM), and GSK343 (1.25 μM) for 24 hr. C, The GR agonist dexamethasone (DEXA, 1 μM), RXR agonist Bexarotene (BEXA, 1 μM) and 6-MP (1 μM) have additive effects on HIV silencing in HC69 cells. HC69 cells were first treated with high dose (400 pg/ml) TNF-α for 24 hr, followed by a 72 hr chase experiment during which the cells were washed with PBS and cultured in fresh media in the presence of placebo (DMSO) or the various NR agonists, alone or in combination. The expression of Nef and β-tubulin in the differently treated cells was analyzed by Western blot. (TIF) [file ppat.1010110.s001.tif]

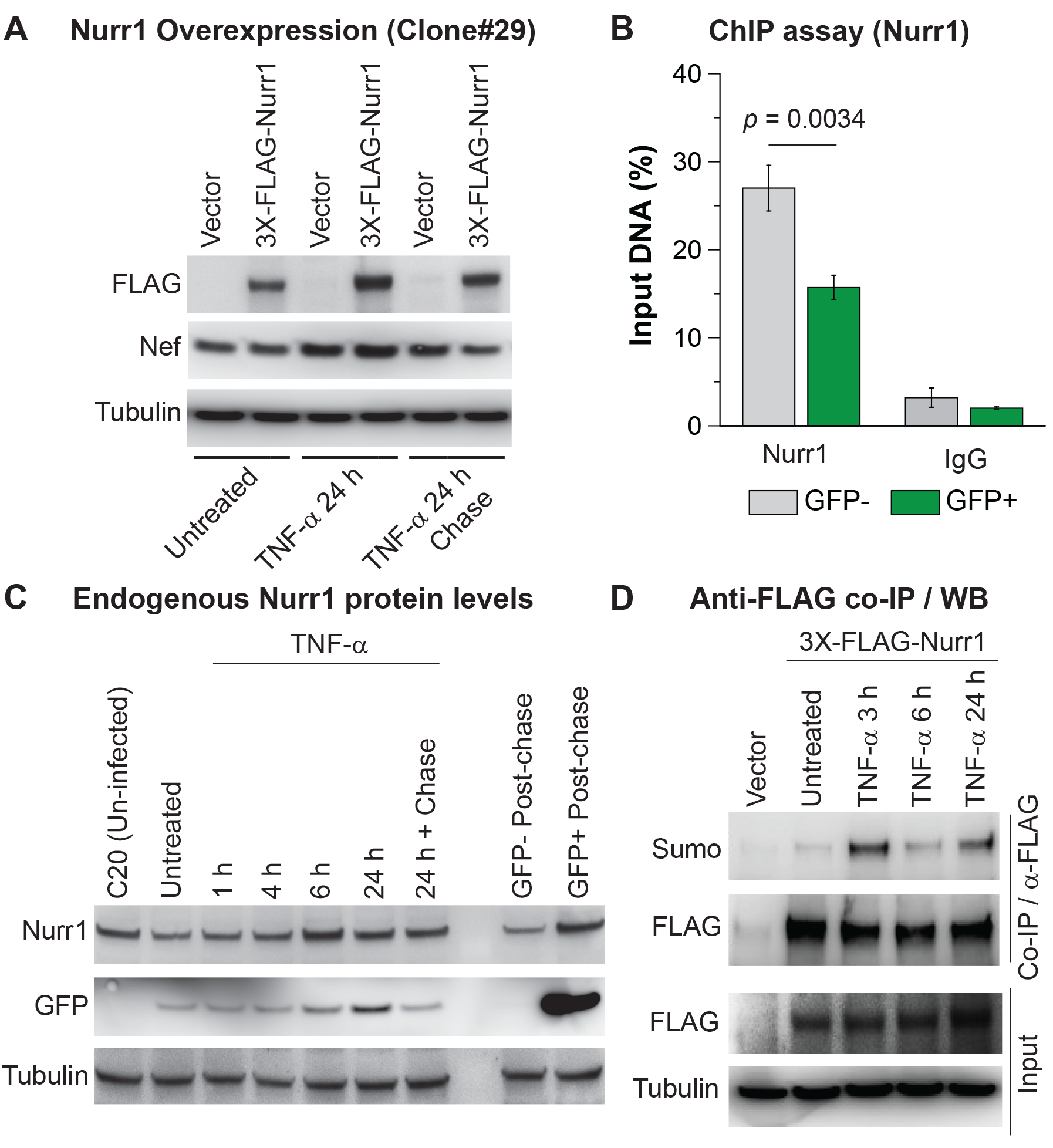

Supplement: S2 Fig — A, Nurr1 overexpression represses HIV in immortalized human microglial cells C20, Clone #29 cells. Western blot detection of Nef, FLAG-tagged Nurr1, and β-tubulin from clone #29 cells that stably express empty vector or 3X-FLAG-Nurr1, which were un-treated, induced with high dose TNF-α (400 pg/ml) for 24 hr, or used in a 48 hr chase after TNF-α stimulation for 24 hr. B, ChIP assay measurement of the levels of Nurr1 in HIV 5’LTR in the GFP+ and GFP- cells. Nurr1 was detected using a mouse monoclonal anti-Nurr1 antibody compared to control IgG. The levels of ChIP products and input DNA were quantified by qPCR by using primers (−390 F, 5’ACA CAC AAG GCT ACT TCC CTG A3’, and −283 R, 5’TCT ACC TTA TCT GGC TCA ACT GGT3’) specific for the Nuc-0 region. The average Nurr1 levels and error bars were calculated from triplicate technical qPCR replicates. C, Impact of TNF-α on endogenous expression of Nurr1. Western blot detection of Nurr1, GFP, and β-tubulin in HC69 cells at different time points post TNF-α stimulation, or in the GFP+ cells and the GFP- cells collected by cell sorting at the end of chase. D. Nurr1 undergoes sumoylation in microglial cells upon TNF-α stimulation. HC69 cells expressing empty vector or 3X-FLAG-Nurr1 that were stimulated with high dose (400 pg/ml) TNF-α for various time points. Total protein lysates from the differently treated cells were used for a co-immunoprecipitation (co-IP) experiment with anti-FLAG M2 magnetic beads (Millipore Sigma, Cat# M8823). The co-IP products were then subjected to Western blot analysis using the anti-FLAG antibody (Fig 3B) to detect 3X-FLAG-Nurr1 and a rat anti-SUMO antibody (BostonBiochem, Cat# A-714) to detect sumoylated Nurr1. The levels of β-Tubulin in the input protein samples were used as loading controls. (TIF) [file ppat.1010110.s002.tif]

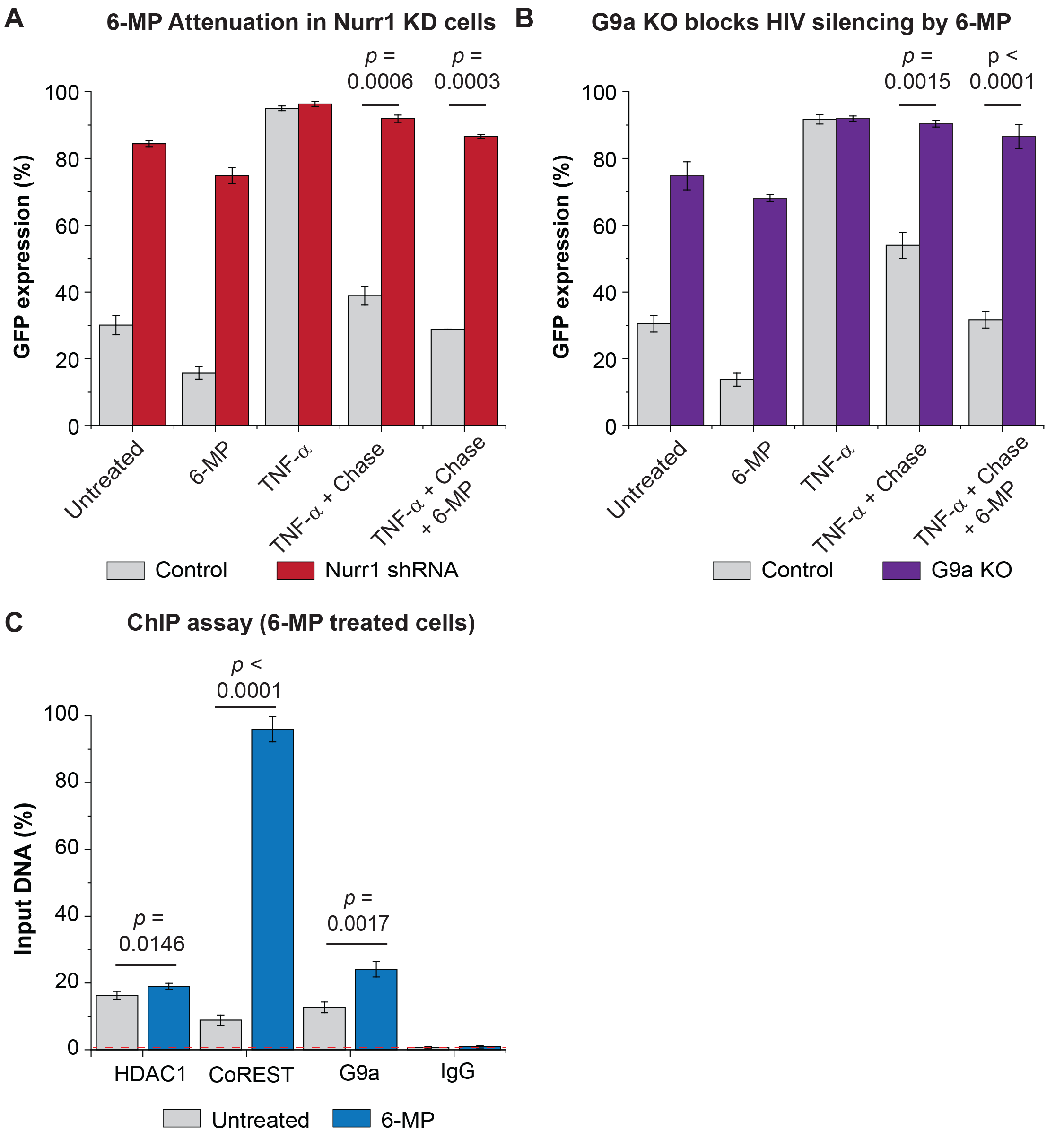

Supplement: S3 Fig — A, GFP expression levels measured by flow cytometry in HC69 cells expressing control shRNA or Nurr1 shRNA. Cells were either untreated or treated with 1 μM 6-MP for 48 hr, induced with high dose (400 pg/ml) TNF-α for 24 hr, and the chased in the absence or presence of 1 μM 6-MP for 72 hr after TNF-α stimulation for 24 hr. Data was calculated using three technical replicates. B, GFP expression levels measured by flow cytometry from HC69 cells expressing empty KO vector or G9a specific guide RNAs (G9a KO) that were treated and assayed as described in A. C, ChIP assay showing relative levels of HDAC1, CoREST, and G9a at the HIV LTR in HC69 cells that were untreated or treated with1 μM 6-MP for 48 hr. Primers were specific for the Nuc-0 region. Data was calculated using three technical replicates. (TIF) [file ppat.1010110.s003.tif]

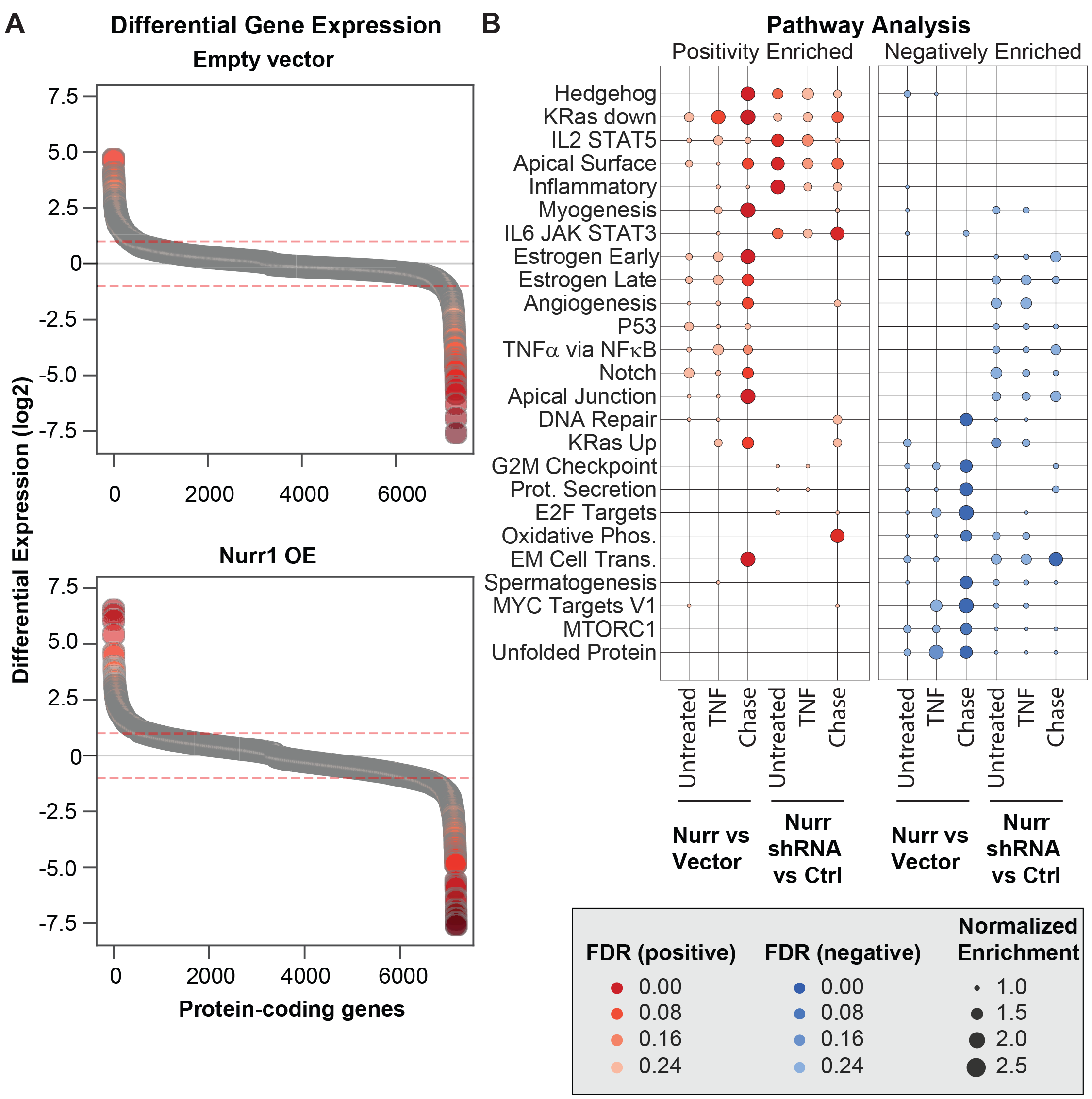

Supplement: S4 Fig — A, Patterns of differential gene expression during the chase step at 48 hr post TNF-α stimulation in vector-infected (top) and Nurr1 overexpressing (Nurr1 OE, bottom) cells. Dotted lines indicate the two-fold cut off level. B, Pathway analyses of Nurr1 overexpression at baseline, during TNF-α stimulation, and following the recovery period after TNF-α stimulation. The identities of specific highly enriched pathways are shown on the Y axis, and the comparisons are shown at the bottom. The color and size of circles correspond to statistical significance, as shown by FDR, and normalized enrichment values, respectively. Positive and negatively enriched pathways are shown in the left and right plot, respectively. (TIF) [file ppat.1010110.s004.tif]

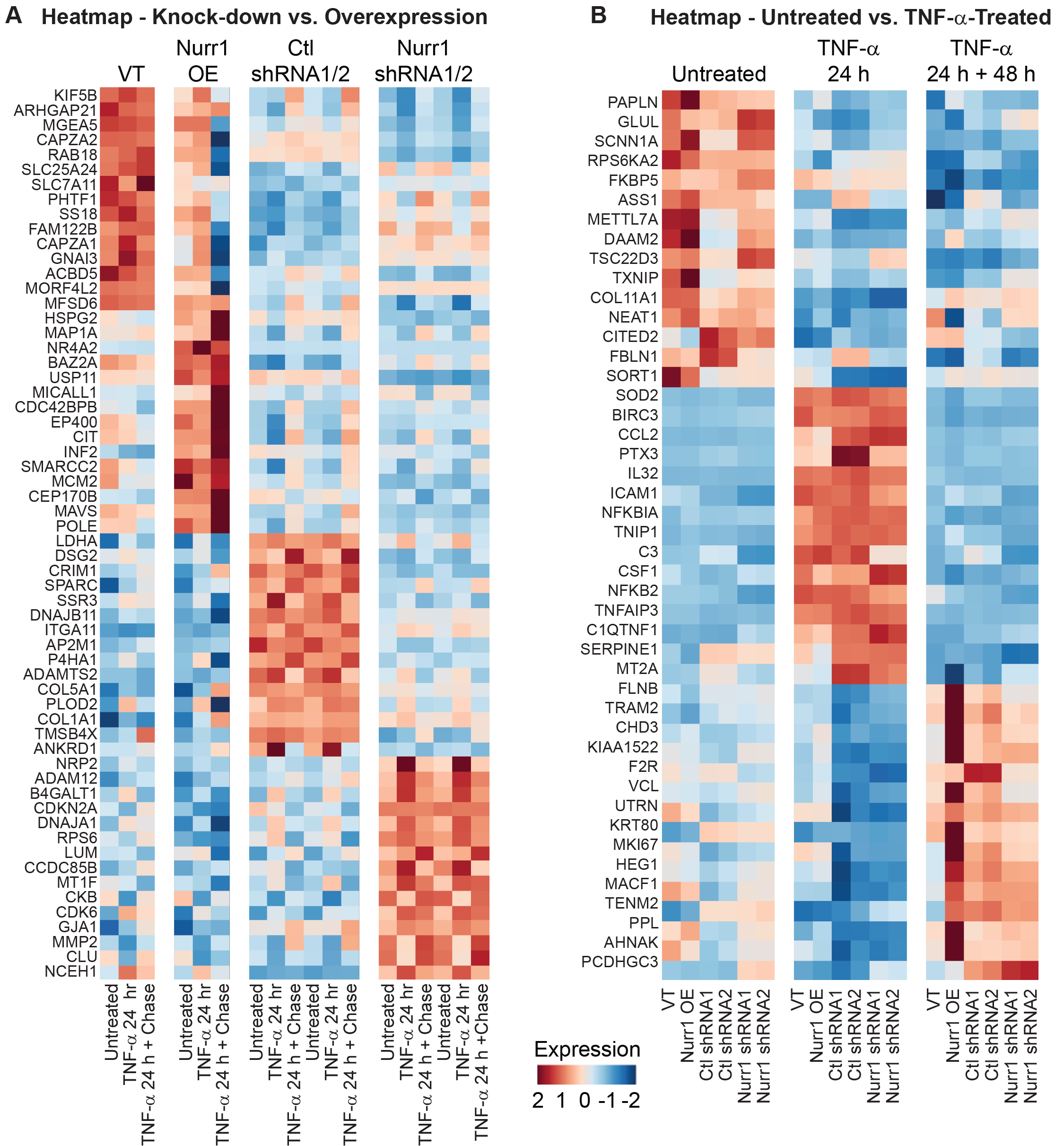

Supplement: S5 Fig — A, Heatmaps representing top 15 gene markers for each treatment group. Statistically-significant (p < 0.001) differentially expressed genes were determined using the Wilcoxon rank-sum test reflecting the impacts of Nurr1 OE by comparing the control cells HC69-3X-FLAG-vector (VT) with Nurr1 overexpressing cells HC69-3X-FLAG-Nurr1 (Nurr1 OE), as well as the impacts of KD by comparing the HC69-control shRNA1 and control shRNA2 (Ctl shRNA1/2) cells with HC69-Nurr1 shRNA1 and shRNA2 (Nurr1 shRNA1/2) cells, respectively. Various cell lines were cultured in the absence (untreated) or presence of high dose (400 pg/ml) TNF-α for 24 hr. In addition, cells were given 48 hr chase after stimulation with high dose (400 pg/ml) TNF-α for 24 hr and subsequent withdrawal. B, Heatmaps showing top 15 gene transcript markers in samples from panel A rearranged according to their status of treatment with TNF-α. The most enriched gene transcripts as the result of Nurr1 overexpression or KD are listed in columns to the left. The color-coded expression pattern of each gene transcript is shown in a heatmap to the right. (TIF) [file ppat.1010110.s005.tif]

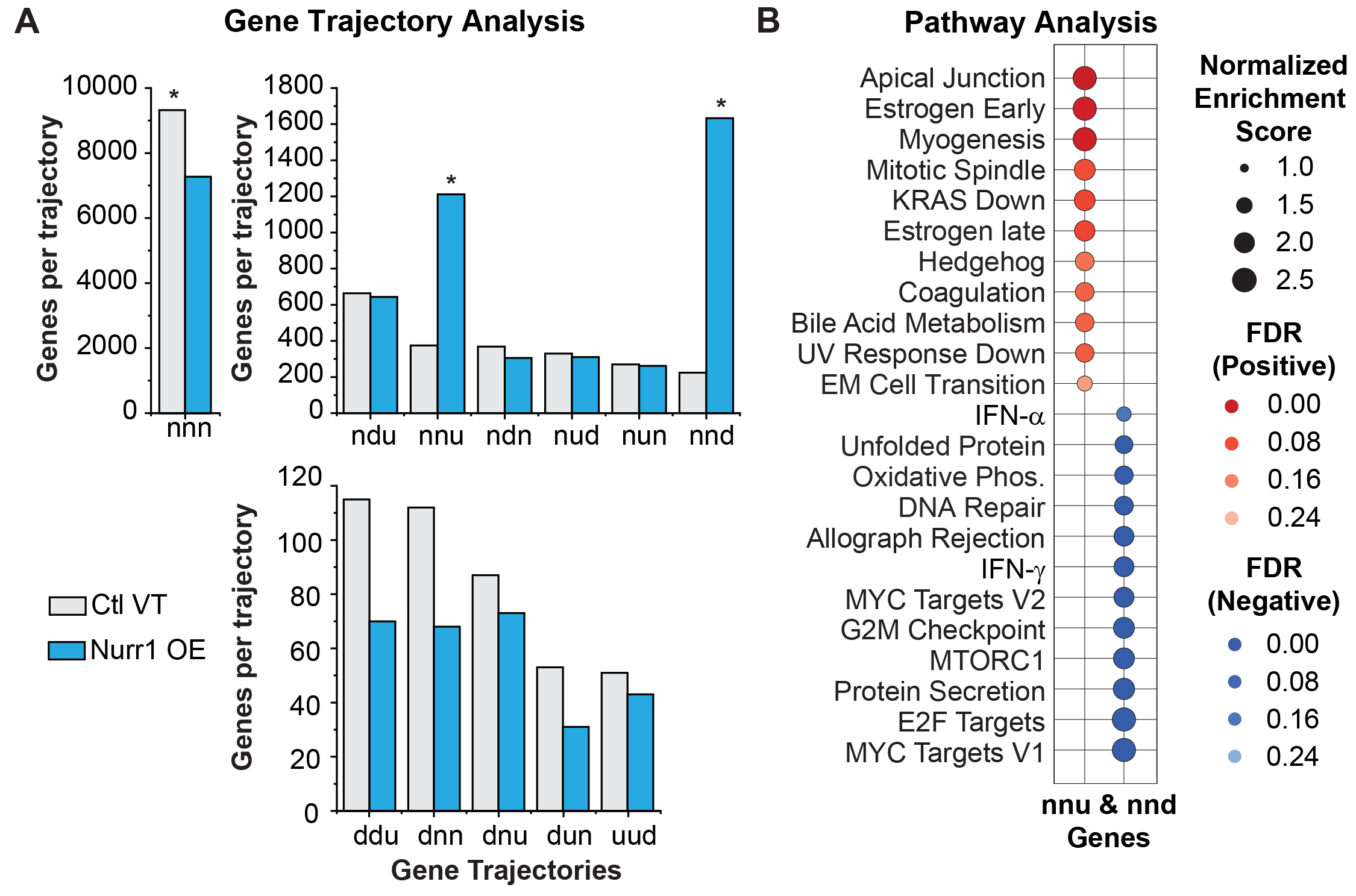

Supplement: S6 Fig — A, Identification of genes selectively altered as a result of Nurr1 overexpression (Nurr1 OE), compared to the control empty vector (Ctl VT) cells, by trajectory analysis. Genes that are unaltered (n), downregulated (d) or upregulated (u) were identified during the activation and the chase steps and were clustered into families with similar profiles. The total number of genes in each category is indicated for both the control and Nurr1-overexpressing cells. Note that the major differences in the gene expression profiles are seen in genes that are either upregulated or downregulated during the chase (highlighted by asterisks). To enable the visualization of the trajectories with low, medium and high membership, the X axis for each group is shown separately. B, Pathway analysis using the Hallmark gene lists of the MSigDB database was performed on non-TNF-α-responsive genes that are exclusively altered in expression during the chase step in Nurr1 overexpressing cells, corresponding to genes which follow nnu and nnd trajectories in Nurr1 cells and an nnn trajectory in control cells (see S7 Fig). The identity of each pathway is shown to the left, and the direction of enrichment (+ or -) is shown at the bottom. The color and size of circles corresponded to statistical significance, as shown by FDR, and normalized enrichment values, respectively. (TIF) [file ppat.1010110.s006.tif]

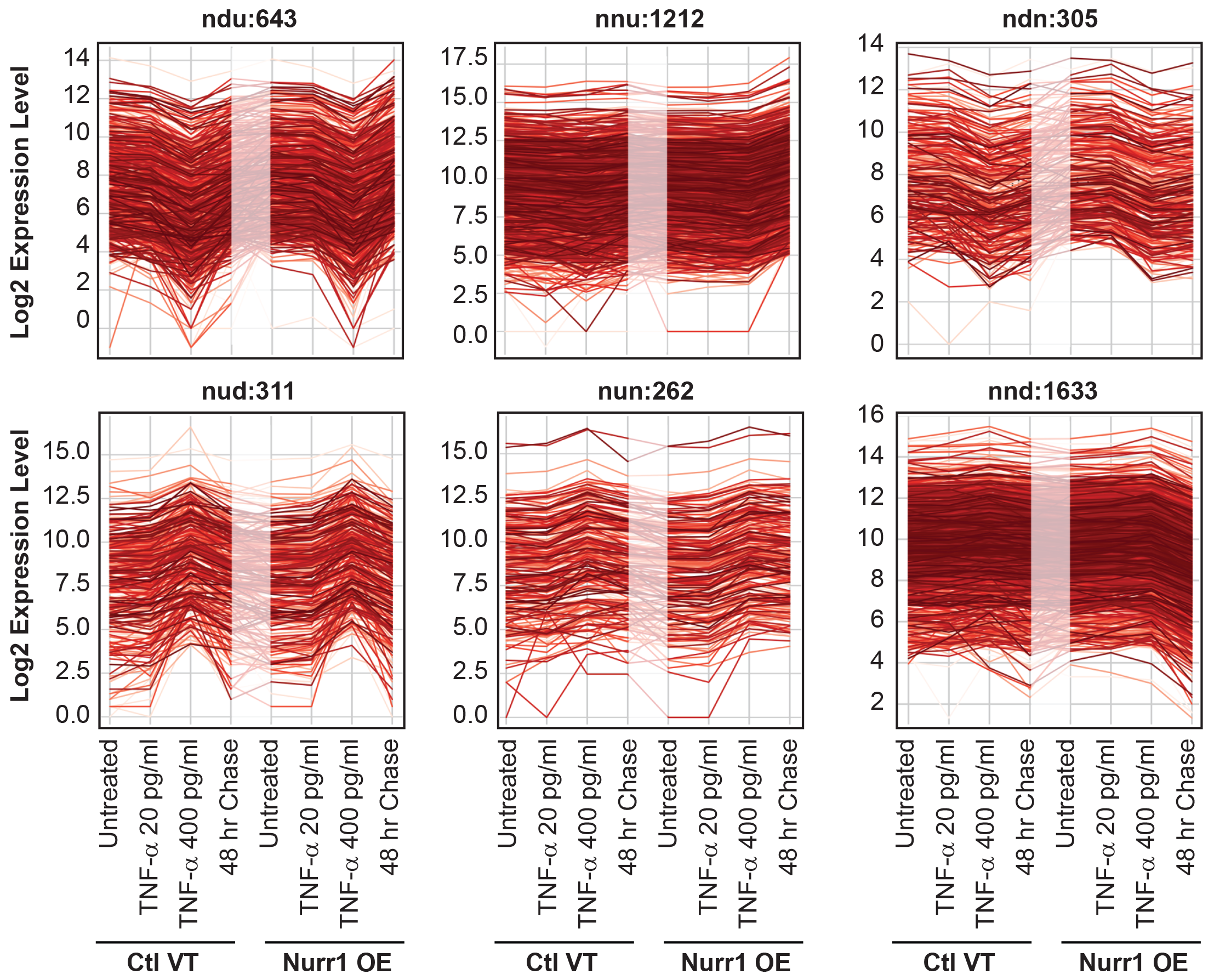

Supplement: S7 Fig — Trajectories of genes after stimulation with low dose (20 pg/ml) and high dose (400 pg/ml) TNF-α for 24 hr and following a 48 hr recovery (chase) after high dose TNF-α stimulation for 24 hr and subsequent withdrawal in the Nurr1 overexpression cell line HC69-3X-FLAG-Nurr1 (Nurr1 OE) were shown. Trajectories of the same genes in the control cell line HC69-3X-FLAG-vector (Ctl VT) were also shown, with a semi-transparent line connecting identical genes between the control and Nurr1-overexpressing sides of each graph. Each line represented a gene, and the Y axis values indicated the log2 expression levels. The number of genes showing each trajectory in Nurr1-overexpressing cells was shown on top. Genes that showed no change, were up regulated, and down regulated in statistically significant manner (FDR<0.05, fold change>2) were indicated with the letters n, u, and d respectively. Grouping of the different trajectories was based on gene responses during stimulation with low dose (Step 1) and high dose (Step 2) TNF-α and the recovery time after TNF-α stimulation and subsequent withdrawal (Step 3). For instance, the group of genes marked “ndu” represented genes that were not significantly changed in response to stimulation with low dose TNF-α but were down regulated with high dose TNF-α stimulation and then up regulated during the recovery (chase) period. (TIF) [file ppat.1010110.s007.tif]

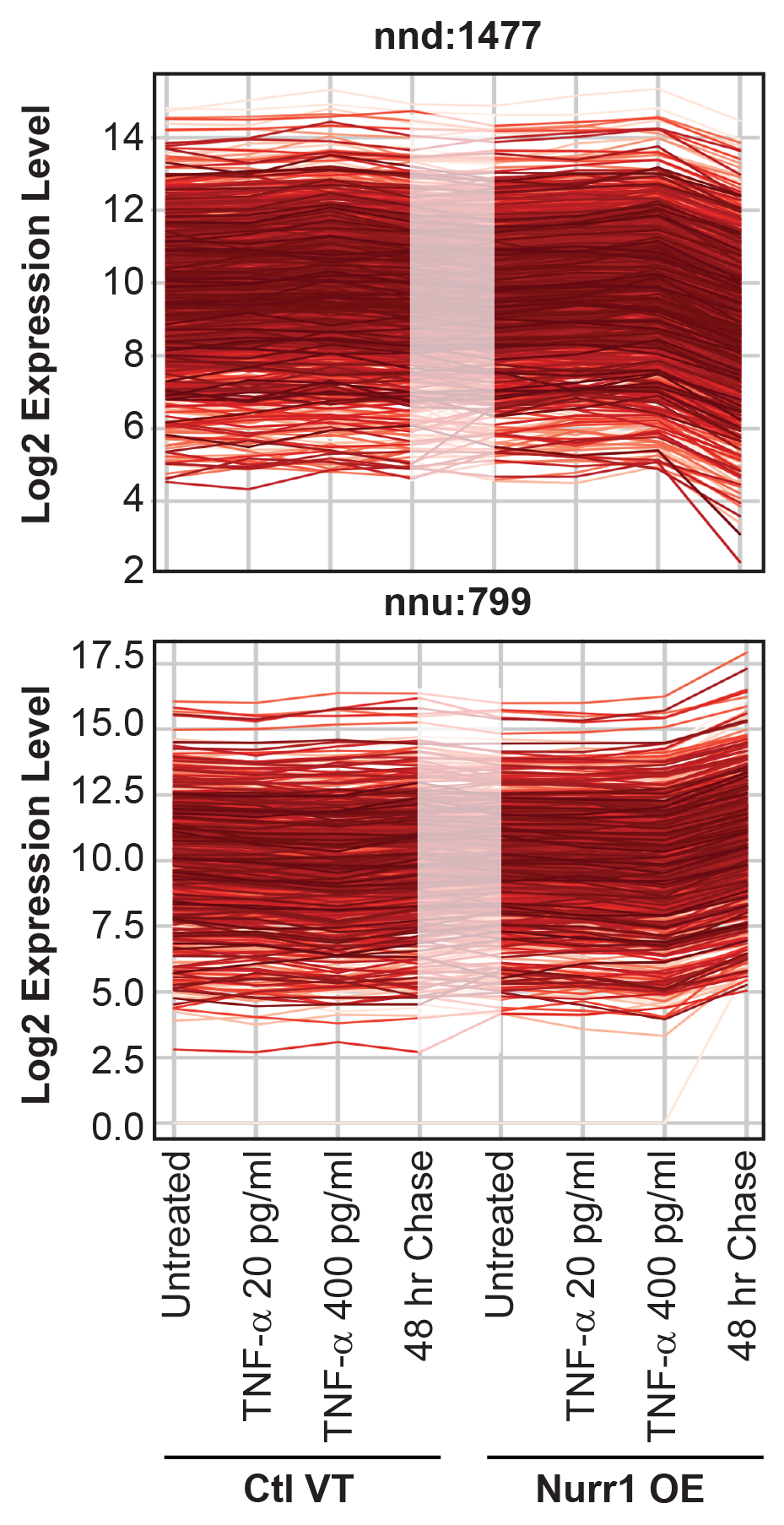

Supplement: S8 Fig — Genes that showed a different trajectory after TNF-α stimulation for 24 hr and following a 48 hr recovery period between HC69-3X-FLAG-vector (control) and HC69-3X-FLAG-Nurr1 cells were identified and groups containing over 100 genes were graphed. Each line represented a gene and a semi-transparent line connected identical genes between control and Nurr1-overexpressing sides of each graph. The Y axis indicated the expression level of each gene throughout the trajectory. Grouping of genes with no statistically significant changes in expression (n), up regulated (d), or down regulated (d) in the three steps of reactivation and chase experiment. (TIF) [file ppat.1010110.s008.tif]

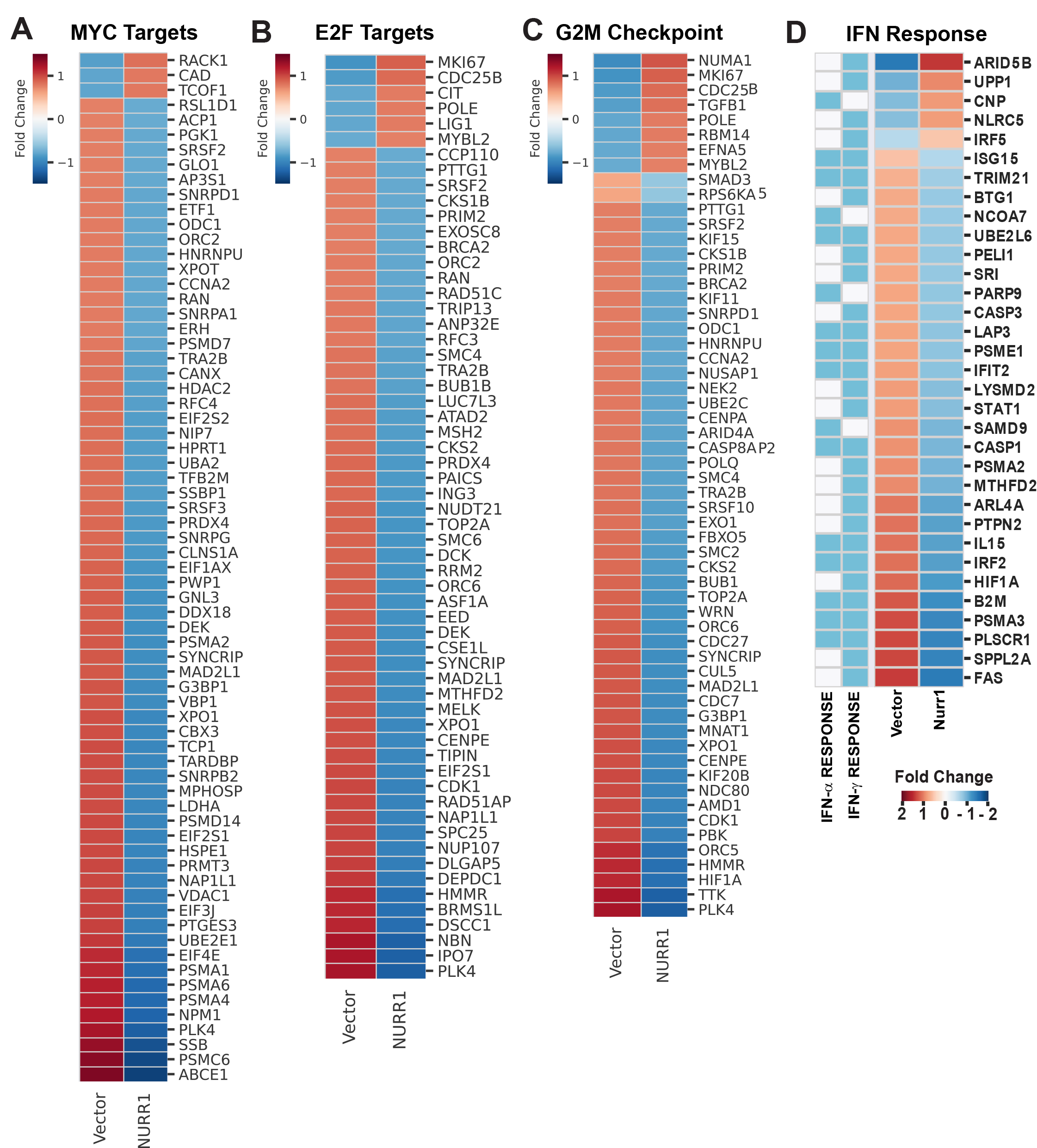

Supplement: S9 Fig — Heatmaps showing the genes involved in the top negatively enriched pathways in Nurr-1 overexpressing cells. A, MYC Targets. B, E2F Targets. C, G2M Checkpoint. D, IFN Response. The values shown in the heatmap correspond to the level of differential expression between Nurr1 overexpressing cells (marked as “Nurr1”) versus vector-infected control cells (marked as “Vector”) during the chase step. The identities of the plotted pathways and genes involved in the pathways are shown on the top and to the right, respectively. (TIF) [file ppat.1010110.s009.tif]

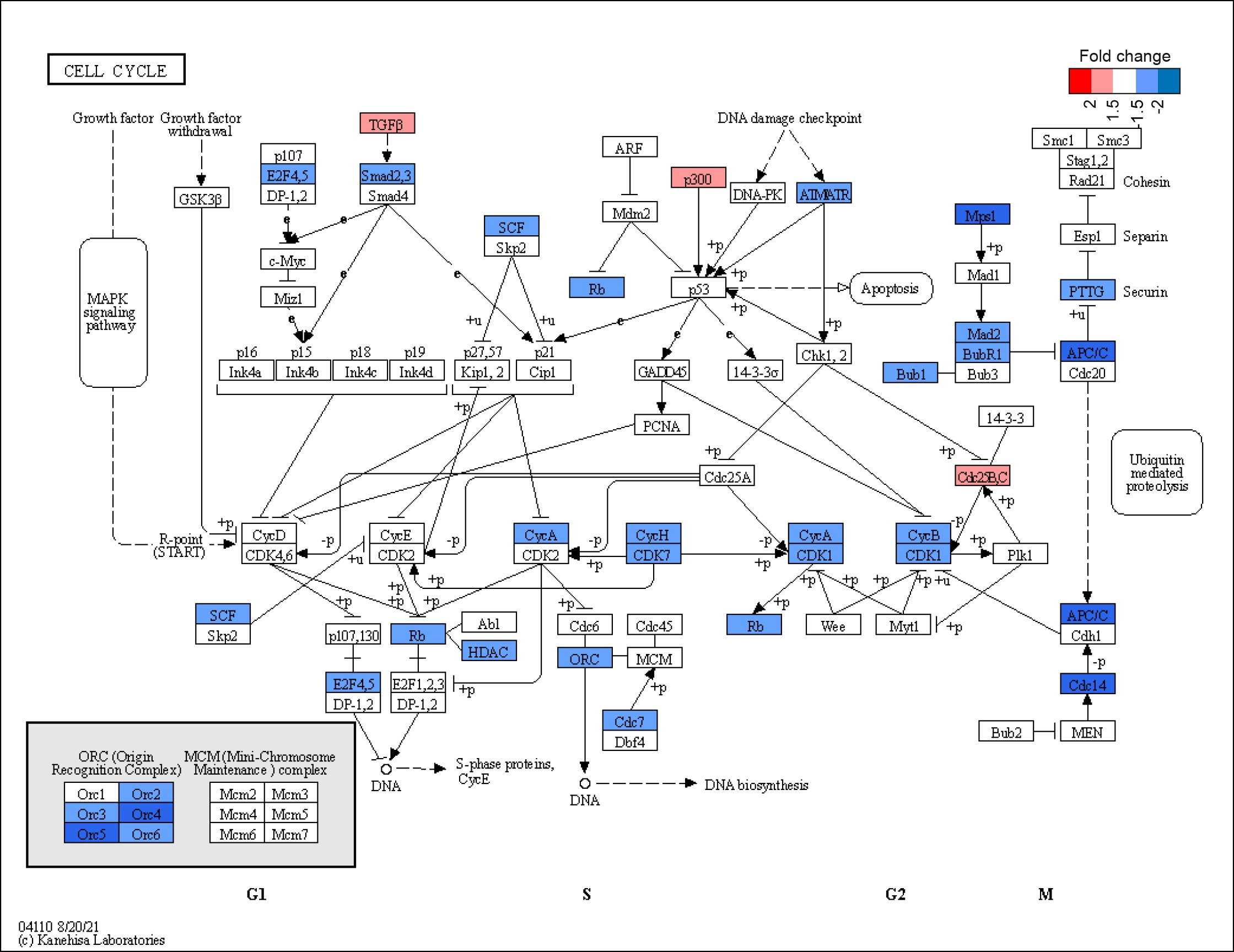

Supplement: S10 Fig — Genes that exclusively change in expression during the chase step only in Nurr1 overexpressing (Nurr1 OE) cells (see S6 Fig) were superimposed on the KEGG cell cycle graph [120]. The color bar on the top right indicates the level of differential expression for each gene in Nurr1 cells during the chase step. (TIF) [file ppat.1010110.s010.tif]

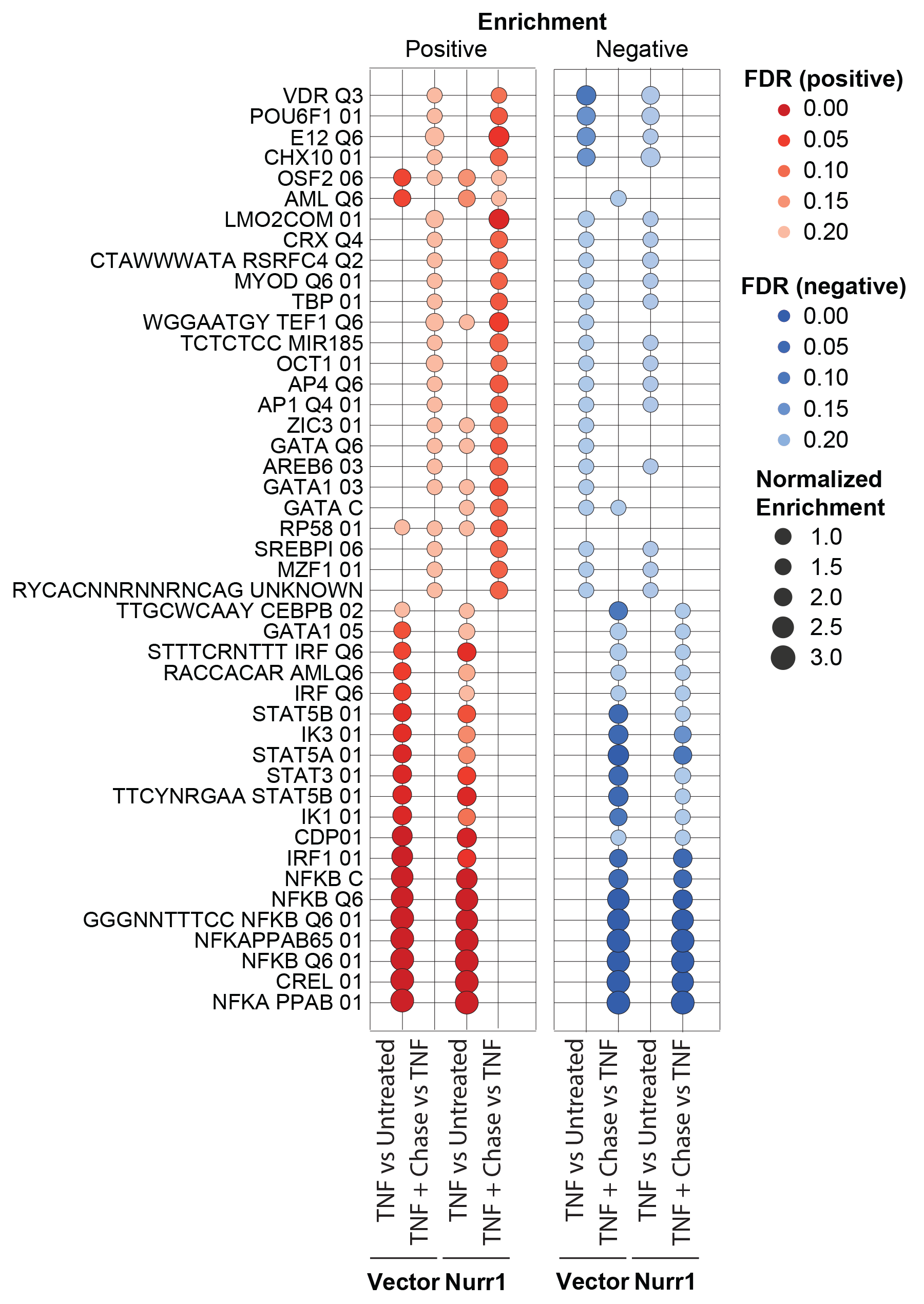

Supplement: S11 Fig — The most enriched transcription factor binding motifs in proximity of the promoters of differentially expressed genes are shown. The size of the circles indicates the level of enrichment, while the color intensity reflects the statistical significance as shown by FDR. Positively- and negatively-enriched motifs are shown after each treatment (shown at the bottom) in the left and right panel, respectively. The identity of each motif, as annotated in the C3 lists of the MSIGDB database, is shown to the left. (TIF) [file ppat.1010110.s011.tif]

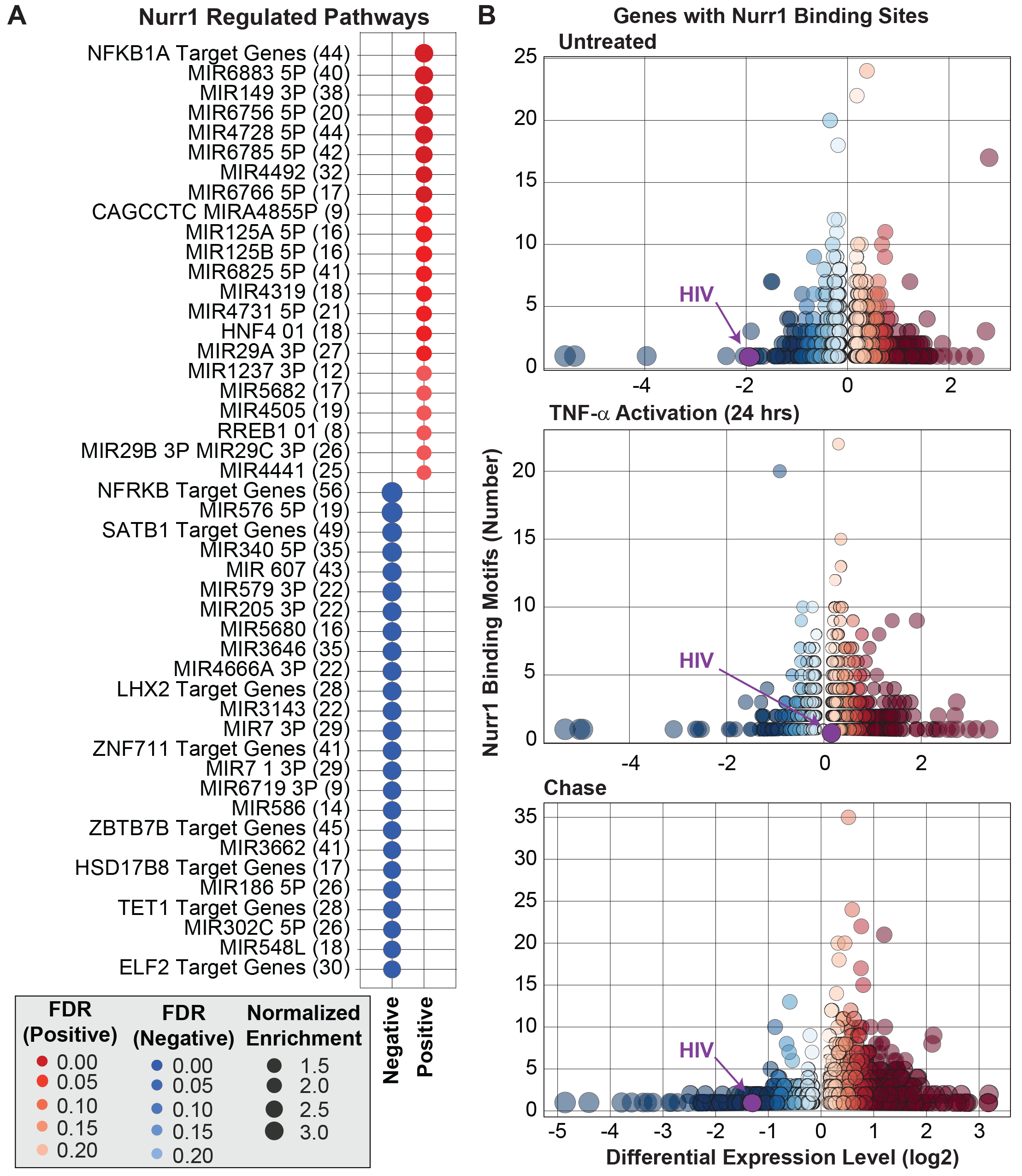

Supplement: S12 Fig — Genes with NURR1 binding sites within 2000 nucleotides of their locus were identified using the NURR1 ChIP-seq studies reported by the ENCODE project. A. Pathway analysis of genes that were negatively enriched or positively enriched in Nurr-1 overexpressing cells during the chase step. B. Volcano plots showing the distribution of Nurr-1 dependent differentially expressed genes carrying one or more Nurr1 sites. Top: Untreated cells. Middle: Cells activated with 400 pg/ml TNF-α for 24 hr. Bottom: Cells chased after activation for 72 hrs. HIV is highlighted in purple. Note that the majority of Nurr1 binding site-containing genes were upregulated as a result of Nurr1 overexpression. However a fraction of genes, including HIV were downregulated under these conditions. (TIF) [file ppat.1010110.s012.tif]
