# Supplementary material for: Recruitment of the CoREST transcription repressor complexes by Nerve Growth factor IB-like receptor (Nurr1/NR4A2) mediates silencing of HIV in microglial cells
Source: PLoS Pathog. 2022 Jul 7;18(7):e1010110. doi: 10.1371/journal.ppat.1010110 (PMC9295971; doi:10.1371/journal.ppat.1010110)
Supplement: Attachment — Submitted filename: Response to Reviewers.docx [file ppat.1010110.s014.docx]

**PPATHOGENS-D-21-02310**

**The Nerve Growth Factor IB-like Receptor Nurr1 (NR4A2) recruits CoREST transcription repressor complexes to silence HIV following proviral reactivation in microglial cells.**

**Responses to reviewers’ comments**

We thank the reviewers for their detailed comments, which helped us to greatly strengthen the manuscript. The reviewers’ comments are in black text. Our responses are in blue text.

**Part I - Summary**

**Reviewer #1:** The paper by Ye et al explores the ability of the nuclear receptor, Nurr1, to repress HIV-1 transcription in the context of microglia. The relevance of these studies is it addresses mechanisms that may contribute to the establishment and maintenance of HIV latency in microglia, a reservoir in the brain, as well as potential pathways that may contribute to CNS inflammatory comorbidities. Evidence presented that supports the conclusion that Nurr1 represses HIV transcription includes Nurr1 agonist repressing transcription in immortalized human microglia cells and iPSCs (the iPSC derived microglia maybe the strongest and most provocative data), and overexpression and knockdown experiments. Furthermore, repression was associated with recruitment of a CoREST associated repressor complex. Most experiments were performed with TNF-α and assessing how Nurr1 influenced the ability of this cytokine to induce HIV-1 transcription in microglia. These findings provide an interesting foundation for understanding the control of HIV in microglia by Nurr1 but it also rises many questions. For example, is ligand dependent repression similar to the silencing seen in response to TNF-α? How is Nurr1 regulated? Does Nurr1 bind DNA or mediate its activity through a trans-repression mechanism? Furthermore, although interesting, the transcriptome data seems to be a tangent and distracts from the focus on Nurr1 transcriptional mechanisms. Some specific comments are below.

We appreciate this reviewer’s comments and concerns about adding additional mechanistic details about how Nurr1 represses HIV transcription. In response, we have conducted a series of additional experiments demonstrating that:

1. The Nurr1 agonist 6-MP silences HIV through the CoREST repressor complex, which is the same mechanism used under condition of Nurr1 overexpression to silence HIV after TNF-α stimulation (**New S3 Fig**). If the referee forgives a somewhat pedantic point of clarification, Nurr1 is an orphan receptor that does not have a known natural ligand, but it does respond to agonists such as 6-MP.
2. Trans-repression of HIV is triggered by Nurr1 phosphorylation induced by NF-kB inducible kinase (NIK) following stimulation by factors such as TNF-α and LPS (**New Fig 10**). We showed by using shRNA knockdowns of NIK that this kinase is required for efficient HIV silencing (**New Fig 10**).
3. Nurr1 binds directly to the HIV provirus at HIV LTR. This contributes to repression of basal HIV expression. We showed that the HIV LTR contains a canonical Nurr1 binding site (**New Fig 5A**). We also demonstrated direct binding of Nurr1 to HIV LTR *in vivo* by ChIP assays (**New Figs 5 to 8**) and mutagenesis of the Nurr1 protein DNA binding motif (**New Fig 5**).
4. Nurr1 binding to HIV provirus is essential for silencing active HIV after TNF-α stimulation. This post-stimulation silencing of HIV by Nurr1 requires its own phosphorylation and subsequent recruitment of the CoREST transcription repressor complex for epigenetic silencing (**New Figs 6, 7, 8 & 9**).

We do not agree that the RNA-Seq experiment is distracting. Not only does this provide the most rigorous way to evaluate HIV transcription and alteration of Nurr1 expression due to overexpression or knockdown, it also lets us evaluate parallel effects on cellular genes. The results thus provide a complementary evidence for a crucial role of Nurr1 in reversing active HIV to latency and driving activated microglial cells towards homeostasis. Nonetheless, we have simplified our discussion of the transcriptomic analyses for the more biologically orientated readers in the main text and now have a single summary volcano plot and pathway analysis figure (**New Fig 12**). A comprehensive set of data underpinning this analysis is now in the Supplemental materials (**New S4 to S12 Figs**).

**Reviewer #2:** In the manuscript “The Nerve Growth Factor IB-like Receptor Nurr1 (NR4A2) recruits CoREST transcription repressor complexes to silence HIV following proviral reactivation in microglial cells”, Ye et al and colleagues report on their investigation of the Nerve Growth Factor IB-like nuclear receptor Nurr1 (NR4A2 and its role in restricting HIV transcription in microglia cells. The authors’ major finding is that Nurr1 plays an important role in shutting down HIV transcription in microglia cells via the recruitment of the CoREST/HDAC1/G9a/EZH2 transcription repressor complex to the HIV promoter. Using transcriptomic analyses, Ye et al also report that Nurr 1 downregulates several cellular genes associated with inflammation, cell cycle and metabolism to promote HIV silencing and microglia homeostasis.

Major Comments

The authors have made some important observations about the role of Nurr1 in HIV transcription in microglia cells. The manuscript is clearly written and provides partial explanation for the silencing of HIV transcription in microglia following spontaneous reactivation that the authors observed in their previous work. However, there are some limitations to this study. Namely, all the mechanistic work was performed using a transformed microglia cell line. Understandably, accessing brain microglia from ART-treated individuals is impossible, but newly established resources such as the Last Gift Study (Rawlings SA et al, PMID: 34257439) could serve as a post-mortem source of well-preserved microglia cells from ART-infected people to at the very least confirm some of the observations made with agonists and epigenetic drugs. The iPSC derived human microglial cells infected in vitro with a reporter virus while provide some information, still lack the nuances of microglial cells isolated from HIV-infected individuals. Additional points that should be addressed are listed below.

We understand the reviewer’s desire to see results from microglial cells obtained from ART-treated HIV patients but unfortunately this is really not feasible. The viability of cells obtained from autopsy patients is limited and they are stressed due to hypoxia leading to problems in even generating scRNA-Seq data, let alone recovering viable cells and performing subsequent experimental manipulations! Furthermore, in the Last Gift study, cART has been removed prior to death, so this becomes a model for active viral replication and is not representative of the clinically relevant status of HIV in HAND. We believe the use of immortalized microglia and iPSC-derived microglia provides the best experimental systems available to study HIV transcription regulation in these cells.

1. The HC69 represents a clonal population of cells with the same integration site. Would the authors conclusion hold true in primary microglia cells isolated from ART-treated individuals where there is likely to be multiple integration site in the microglial cell population?

Our new experiments demonstrate that there are only minor variations in HIV silencing efficiency among different clonal cell lines by Nurr1 ligand 6-MP and/or Nurr1 overexpression. We have now confirmed our results in a wide range of clones (**New Fig 2C and S2 Fig A**) and iPSC-derived microglial cells (**New Fig 11**). Therefore, the effect of Nurr1 on HIV silencing seems to be ubiquitous.

We disagree with the referee’s concern on multiple integration sites commonly seen in microglial cells from patients. Even in T-cells most cells have single viral integrants and we are not aware of any evidence for frequent multiple integrants in microglial cells. Indeed, we think that this is unlikely since the virus titers in the CNS are low limiting opportunities for multiple co-infections. In any case, the Nurr1 mechanism is LTR specific and there is also no reason to think that it does not operate independently of integration sites. For example, mixed populations of cells are repressed just as efficiently as isolated clones (**New Fig 2C**). The difference between clones are generally due to changes in basal expression and do not effect post-activation silencing, which is the main role played by Nurr1.

2. The authors concentrated on Nurr1, however, the western blot analysis in figure 3D would also suggest at least a partial role for Nur77 and Nor1 in HIV silencing.

We agree that Nur77 and Nor1 can play a partial role in HIV silencing. Nor1 inhibits HIV reactivation by blocking NF-κB recruitment, while Nur77 mediates silencing by engaging the glucocorticoid receptor (GR). However, their effects are not as robust as Nurr1. We plan to address the roles of these two nuclear receptors in a follow-up manuscript. It would make this paper overly long and complex if we describe all three mechanisms of action of all three receptors.

3. Figure 8A, as these epigenetic drugs are reported to induce HIV expression at least in T cells, an important control to include is the effect of these compounds on cells that have not been treated with TNF-α. Are they simply reactivating silent HIV genomes?

The epigenetic drugs do reverse HIV latency in microglia, as we have previously described for HDAC inhibitors. We have now added data showing that they can induce latency reversal, as requested (**New S1 Fig B**). However, we do not see why this detracts from the point we are making that Nurr1 helps to recruit epigenetic silencing complexes during the chase. This model was reinforced by the CoREST KD and EZH2 and G9a KO data shown originally. We have added to these data by showing that G9a KO blocks HIV silencing by 6-MP (**New S3 Fig B**) and ChIP data showing enhanced histone methylation in Nurr1 overexpressing cells (**New Fig 8**).

Reviewer #3: Ye et al describe the activity of Nurr1 in suppressing HIV following reactivation in microglial cells. The authors demonstrate this using Nurr1 overexpression, knockout cells, and treatment with the Nurr1 agonist 6-MP. They further demonstrate the mechanism of action is through the CoREST repressor complex. Given most of these experiments are performed in a latent cell line model, the authors also crucially demonstrate HIV suppression in an infection model using iPSC-microglial cells. The authors data also hints at an anti-inflammatory activity of Nurr1 following HIV reactivation which has the potential to be very significant given the role of HIV activity in HAND. Overall, this is a very well written paper (albeit dense) with important findings for HIV latency in the CNS, HAND and the CNS reservoir and will be of interest to those in the field.

We thank the reviewer for their positive comments.

**Part II – Major Issues: Key Experiments Required for Acceptance**
**Reviewer #1:**

1. Initial experiments examined repression of spontaneous activation with Nurr1 agonist. It was not clear as to why ligand mediated repression was not explored in greater detail. It also raises questions as to whether the mechanisms of ligand mediated repression and silencing following TNF-α are similar.

We agree this is an important issue and in response have conducted additional experiments:

1. We have shown that the HIV silencing effect of 6-MP is attenuated in Nurr1 KD cells, suggesting that its action is Nurr1 specific (**S3 Fig A**).

2. By conducting ChIP assays, we have demonstrated that 6-MP increases the levels of CoREST, G9a and HDAC1 recruited to the HIV LTR, indicating that 6-MP utilizes the same mechanism as Nurr1 overexpression to silence active HIV following TNF-α stimulation (**S3 Fig C**). Consistent with this notion, we have also found that 6-MP lost its effect in G9a KO cell line, thus further confirming the action mechanism of 6-MP (**S3 Fig B**).

3. In response to the referee’s concern about the initial activation conditions, we have also shown that 6-MP enhances silencing of HIV after an initial activation by LPS, IL-1β & PIC (**New Fig 2D**).

As mentioned above, each of the members of the NR4A subfamily, unlike many other nuclear receptors, do not possess ligand-binding cavities and are immediate early genes whose expression is induced by various stimuli including cyclic AMP, growth factors, inflammatory signals, and hormones. 6-MP is therefore an agonist and not, strictly speaking, a mimic of a naturally occurring ligand.

2. Does overexpression or knocking down Nurr1 alter the kinetics of activation and repression (spontaneous activation or silencing following TNFa treatment)?

Our data showed that Nurr1 overexpression results in reduced Nef and HIV RNA expression in the absence of TNF-α stimulation (**New Fig 3D & E**) and conversely, that Nurr1 KD results in substantially higher levels of Nef and HIV expression even without TNF-α stimulation (**New Fig 4D & E**). In the chase experiments, HIV silencing is significantly enhanced in the Nurr1 overexpressing cells, and significantly inhibited in the Nurr1 KD cells. We have seen similar results during chases between 24 and 96 hrs. Therefore, we conclude that Nurr1 overexpression or KD does not control HIV silencing by altering the kinetics of HIV activation and repression.

Data from both RNA-seq and Western blot analyses indicate that overexpression of Nurr1 only slightly inhibits HIV transcription when cells are induced with low dose TNF-α and has no effect at all when cells are stimulated with high dose TNF-α, suggesting that Nurr1 binding to HIV provirus does repress HIV transcription during reactivation of the latent HIV (**New Fig 3D & E**). However, the repression is not robust enough to overcome strong stimulation. In contrast, the effect of Nurr1 on silencing active HIV after removal of TNF-α stimulation is dramatic. Thus, our data suggest a trans-repression mechanism for post-stimulation silencing of active HIV, which mainly engages the CoREST transcription repression complex.

3. Overexpressed Nurr1 was demonstrated to Co-IP with the CoREST repressor complex. Was overexpression necessary? Could endogenous Nurr1 pulldown CoREST?

Our co-IP experiment with anti-Nurr1 antibody demonstrated that pulldown using endogenous Nurr1 (vector control) also pulled down CoREST (**New Fig 10C**). The new data are consistent with previous co-IP results (**New Fig 6B**). By way of confirmation, the ChIP-Seq experiment measuring factor recruitment (**New Fig 7**) was performed with control cells. Similarly, the ChIP experiment measuring histone modifications (**New Fig 8**) has control and overexpressing cells. We therefore don’t think that overexpression of Nurr1 perturbs its interactions with CoREST and the other members of this complex.

4. The ChIP of CoREST complexes is interesting. The result suggests follow-up experiments to determine if this correlates with repressive histone post-translational marks. Also, it appears CoREST, G9a and EZH2 are associated with and represses promoters HIV in unstimulated cells (Fig 8) begging the question if these complexes display regulated binding during spontaneous activation?

Our previous studies have shown that spontaneous HIV activation in microglial cells is mainly driven by autocrine expression of TNF-α (Alvarez-Carbonell D, Ye F, Ramanath N, Dobrowolski C, Karn J. The Glucocorticoid Receptor Is a Critical Regulator of HIV Latency in Human Microglial Cells. J Neuroimmune Pharmacol. 2019 Mar;14(1):94-109. doi: 10.1007/s11481-018-9798-1. Epub 2018 Jul 10. PMID: 29987742; PMCID: PMC6394485).

To address the reviewer’s points, we have now included additional data from ChIP assays that demonstrate that Nurr1 overexpression and activation with its agonist 6-MP indeed dramatically increase the levels of CoREST and G9a (with the expected lesser impact on HDAC1) resulting in enhanced HIV silencing by promoting histone acetylation and methylations (**New Fig8** and **S3 Fig C**).

5. Nuclear receptors have been shown to activate and repress genes by ligand dependent DNA binding or through trans mechanisms independent of DNA binding. There are no experiments that provide insight into Nurr1 mechanism of action.

We agree that this is a central issue and we have greatly enhanced our discussion of the Nurr1 mechanism of action as follows:

1. We have now shown that there is a Nurr1 binding motif in the HIV 5’LTR (**New Fig 5A**). To further examine Nurr1 binding to HIV chromatin, we generated a DNA binding deficient Nurr1 mutant construct from the original 3X-FLAG-Nurr1 wild type (WT) construct (**New Fig 5B**). We then established HC69 cells lines that express the empty vector, the WT Nurr1, and the mutant Nurr1 respectively. Data from ChIP assays showed that Nurr1 protein levels at the LTR are substantially lower in cells expressing the mutant Nurr1 than cells express the WT Nurr1, thus confirming Nurr1 binding to the HIV LTR (**New Fig 5C**). Consistent with this, HIV silencing in cells overexpressing the mutant Nurr1 is largely blocked, suggesting that Nurr1 binding to HIV 5’LTR is essential for HIV silencing (**New Fig 5D & E**).

2. Our data from ChIP-seq experiments also demonstrate that Nurr1 is recruited to this chromatin region (**New Fig 7B**).

3. We have also defined a new pathway regulating Nurr1 activity in microglial cells due to TNF-a induced Nurr1 phosphorylation by NF-kB inducible kinase (NIK). We also showed that NIK is required for efficient HIV silencing (**New Fig 10**).

4. We have shown that Nurr1 is a substrate for sumoylation, which may explain how it is removed from HIV LTR (**New S2 Fig D**).

**Reviewer #2:**

1. Line 161, the authors mentioned that the substantial decrease in GFP+ observed in the chase experiments is unlikely due to GR (glucocorticoid receptor )-mediated HIV silencing as the cells were not cultured in the presence of dexamethasone, a ligand of GR. What were the culture conditions for the cells? This is missing in the methods section. The presence of fetal bovine serum (FBS) could be a potential source of cortisol (GR ligand) to which the cells are exposed to.

In all experiments in this paper using either the empty control vector or 3X-FLAG-Nurr1, or cells with and without treatment with 6-MP, we use culture media with 1% FBS, which reduces any potential cortisol in the serum to levels that do not block spontaneous reactivation. As we reported previously under these conditions exogenous DEXA is needed to silence the spontaneously reactivating cells, which are driven by autocrine expression of TNF-α (Alvarez-Carbonell D, Ye F, Ramanath N, Dobrowolski C, Karn J. The Glucocorticoid Receptor Is a Critical Regulator of HIV Latency in Human Microglial Cells. J Neuroimmune Pharmacol. 2019 Mar;14(1):94-109. doi: 10.1007/s11481-018-9798-1. Epub 2018 Jul 10. PMID: 29987742; PMCID: PMC6394485). Thus, the impact of potential cortisol from FBS is well controlled by culturing all cell lines under identical conditions and does not affect our conclusion.

2. What is the effect of toxicity of the agonists and epigenetic drugs used on some of the observations? The authors should provide toxicity data on the microglia cells

To address this valid concern we have included control toxicity experiments (**New S1 Fig A – C**). There is no significant toxicity for the compounds at the concentrations we used in our experiments.

3. Line 383 and figure 7, there is really no strong evidence to suggest that G9a and HDAC1 decreased at the promoter at 4hr post-TNF-α treatment. In fact, this is clearly not the case in the qPCR results. The minor differences in the peaks in the ChiP seq experiment could be due to experimental fluctuations. Did the authors use a normalization approach, i.e spike in a known quantity of a certain DNA to standardized between samples (e.g., ChIP seq spike in normalization by Active Motif). Also, are the scales the same for the read counts (since they are not shown)? How many times were these experiments performed?

We agree with the reviewer’s comments and have made changes to the text and revised Figure, which also includes Nurr1 (**New Fig 7**). Although we did not use a spike in control, we did normalize for input DNA after our HIV specific enrichment procedure, which is described in the Materials and Methods. The read counts axes were consistent for each antibody but, of course, they vary between antibodies and we give the values in the revised Fig legend. The experiments were performed multiple times, and cross referenced to qPCR results, but because of the nature of the data only a representative experiments is shown.

4. In fact in many of the figures, it is not cleared how many times the experiments were repeated (e.g. western blot analyses data). The number of independent experiments performed to demonstrate reproducibility should be clearly stated for all the figures (not just the replicates). Also, the type of statistical tests performed should be indicated in both the figure legends and a statistical analyses plan should be included in the methods.

We have now described statistical methods and provided *p*-values and replicate numbers in the Figure legends. In **New Fig.1D** we show data for the 6-MP effect from 9 independent experiments performed over a 2-year period. This shows in a very convincing way that the cell system is highly consistent and reproducible. We obviously did not go to these lengths for each experiment, which are a complementary, consistent and interlocking set of data that should be look at in their totality. For individual experiments this type of data really doesn’t require anything more elaborate than simple *p*-value calculations.

**Reviewer #3:**

1. If indeed Nurr1 is silencing HIV transcription, a useful confirmatory experiment would be to sort the GFP+ and GFP- cells at the end of your chase experiment (61% GFP +) and determine Nurr1 expression (RNA, protein) in these two populations. One would expect higher levels of Nurr1 in GFP- cells. This experiment could be performed using the iPSC- infection model.

In response to this interesting comment, we stimulated HC69 cells with TNF-a for different time points and traced Nurr1 expression [Note: iPSC was not used for this experiment due to limitation of cell numbers]. We sorted GFP- and GFP+ at the end of chase as suggested, and measured Nurr1 expression. Surprisingly, we detected slightly higher Nurr1 protein levels in TNF-a induced cells than untreated cells (**S2 Fig C**). We also found higher levels of Nurr1 protein in sorted GFP+ cells than in GFP- cells at the end of chase, suggesting Nurr1 upregulation in cells with active HIV expression. However, data from ChIP assays indicate that the levels of Nurr1 in HIV LTR is higher in GFP- cells than in GFP+ cells (**S2 Fig C**). Thus, it is the amount of chromatin-bound Nurr1, not the overall level of Nurr1 protein in a cell, that regulates HIV expression.

Our transcriptomic analyses also show that the majority of genes with Nurr1 binding sites become upregulated. It is only genes regulating the cell cycle and microglial activation status, along with HIV, which are downregulated (**New Fig 12**).

Furthermore, in total agreement with previous report, we found that Nurr1 is subjected to sumoylation, which increases following TNF-a stimulation in microglial cells (**S2 Fig D**), providing a plausible mechanism to regulate Nurr1 protein levels.

These new results do not contradict our findings involving Nurr1 overexpression or its activation with agonist 6-MP. In HC69-3X-FLAG-Nurr1 cells, Nurr1 is overexpressed before TNF-a stimulation. Compared to control cells, overexpression of Nurr1 or its activation by 6-MP induces larger amounts of CoREST complex to HIV chromatin after TNF-a stimulation, resulting in enhanced HIV silencing.

2. It would be useful to confirm that 6-MP is exerting its effect through nurr1 by performing the 6-MP suppression experiment using the Nurr1 knockout cells. This is especially important given the confirmatory experiments in iPSC were done with 6-MP.

This is a valuable control, which is now present in the supplementary information (**New S3 Fig A**). 6-MP suppression is indeed eliminated in Nurr1 knock down cells. We also extended the experiment to use G9a knockout cells and showed that G9a is also critical for HIV silencing induced by 6-MP (**New S3 Fig B**).

**Part III – Minor Issues: Editorial and Data Presentation Modifications**

Reviewer #1:

1. Figure 1 may not be necessary or use as supplemental information. They have described the cell line in previous experiments and used it to examine glucocorticoid receptors and Dex mediated repression of HIV in previous publications.

We have revised Figure 1 (**New Fig 1**), which now includes the 6-MP results. We think the introduction to the HIV reporter, HIV silencing, and chase design are helpful to the reader and we have retained them in the Figure.

2. The transcriptomic experiments are interesting and indicate a role for Nurr1 microglia but are a tangent to understanding how Nurr1 controls HIV expression. It would also be interesting to include Nurr1 ligand and compare to cells overexpressing Nurr1.

As discussed above, there seems to be a confusion on the part of the referee about ligands for Nurr1, which like the other orphan receptors does not have a known ligand. We have greatly expanded the mechanistic studies presented in the revised manuscript, as described above. We actually feel the transcriptomic data is integral to the story, since we have used it monitor Nurr1 and HIV expression simultaneously and identify subsets of genes that are differentially expressed in response to Nurr1. However, to lessen the pain for the more biologically oriented readers we have placed most of the transcriptomic data in the supplementary information and now have a single simplified Figure in the main text (**New Fig 12**).

We agree that similar RNA-seq analysis should be done to look at the effect of Nurr1 activation by 6-MP. However, we prefer to address this issue in our next follow-up manuscript in which we will assess the individual and combinational effects of 6-MP, DEXA, and BEXA, ligands for Nurr1, GR, and RXR, in a iPSC-derived microglia & neuron co-culture system by conducting scRNA-seq.

Reviewer #2:

3. Line 69 appears to be incomplete: “However, the early studies neglected both the side effects of anti-HIV drugs on neuronal damage which could mask the benefits of reduced HIV expression by cART and the impact of HIV latency on the development of HAND”. Both the side effects of anti-HIV drugs and what else?

We have corrected this ambiguity. Thanks for catching it!

4. As this is still controversial and under intense investigation, can the authors confirm their statement that microglia are “long-lived” reservoirs in ART-suppressed individuals (line 76). The reference provided (Ref 20) does not provide concrete evidence.

We agree that there is little consensus in the field! We revised the introduction and discussion to be more specific that HIV persists in the CNS and that the best candidates for a reservoir are the microglial cells, which have a longer lifespan than astrocytes, and that astrocytes are not infected at high frequencies. We have also added some more appropriate references.

3. In the methods section, instead of randomly listing the antibodies used in the study, to help guide the readers, the antibodies should be listed in accordance with the assay it was used in (e.g. ChiP, immunoblotting, etc.). Are the ChiP antibodies validated for the purpose of chromatin immunoprecipitation for example.

Good suggestion! We have made the appropriate changes.

Reviewer #3:

1. Line 64-65: ART lowers HIV RNA in the brain – the first article is an SIV paper, the second uses samples from PLHIV however the majority of them were not virally suppressed, can we find some better references?

We have added more appropriate references.

2. Line 76-77: given the persistence of HIV in CNS remains a controversial topic, perhaps this assertations could be backed up by primary articles rather than a review article?

As described above we have modified the Introduction and Discussion. We don’t think HIV persistence in the CNS is really that controversial, although patient studies are obviously very limited in their scope and rely on post-mortem samples. The landmines are about which cells are infected and the relative contributions of systemic inflammation, ART and virus on neuropathogenesis. It’s almost impossible to gracefully avoid all of these controversies, but we have done our best to state our position.

3. Figure 1 spontaneous silencing, could this not just be outgrowth of GFP- cells?
Figure 2A: toxicity of 6-MP in cells?

We have not seen substantial differences between GFP- and GFP+ cells in term of cell growth, and in any case the 24 to 96 hr chase conditions we have used are too short for substantial outgrowth to have occurred.

The toxicity data of 6-MP has been added (**New S1 Fig A**).

4. Figure 2B: Intense Nur77 band with 5uM 6-MP, but this doesn’t titrate. Is this an error? Can authors address.

Actually at high concentrations (5 to 20 μM) 6-MP is known to induce expression of Nur77 in microglial cells. We have added a comment and the earlier reference to address this point.

5. Figure 2C: I don’t think this data is really useful or necessary. Also I don’t think you can use nef WB to look at this, should be GFP. If authors want to assess 6-MP synergy then they should do proper synergy experiments and use the GFP reporter rather than nef WB

The GFP reporter HIV expresses Nef and GFP on the same transcript, with the two ORFs separated with an IRES site. As a result, we always see an excellent correlation between protein expression levels of GFP and Nef. However, we typically use GFP for quantification of HIV expression by flow cytometry since this does not require an additional cell permeabilization and staining step. For Western blot analysis, we prefer to measure Nef as a marker for HIV expression since we have good antibodies and it is the native viral marker.

6. Figure 3A: Only Nurr1 shown, perhaps this is better shown with a bar graph of total reads (and include all three genes)?

We think showing how the actual Nurr1 reads map to the gene is highly informative (**Fig 3B**) because it shows the quality of the underlying sequence analysis. But, in any case, the Nurr1 during each step of the chase experiment are presented at a line graph in **Fig 3E**. We showed Nurr77 and Nor1 for the sake of completeness, but do not want to distract attention from Nurr1. As mentioned above we will provide more information about Nurr77 and Nor1 in a follow up manuscript. It was simply too much data and too unwieldy to cover all three receptors in the same manuscript.

7. Figure 3B: Expression of Nurr1 and Nor1 much less than previously seen (figure 2B), even though tubulin bands have higher intensity?

The two blots (now as **New Fig 3B & D**) cannot be compared directly since they were from different experiments with different exposure times but the relative expression levels of the different receptors are consistent.

8. Figure 4A: Enumeration of the magnitude of Nurr1 knockdown is required. The way the data is currently presented does not give this sense.

Nurr1 shRNA resulted in a 2.6 fold reduction of Nurr1 mRNA. We added the Nurr1 KD information in the figure legend.

9. Figure 5: At what stage during the chase is the RNAseq performed, this is not made clear?

We have moved Figure 5 to supplemental information (**New S4 Fig**). The RNA-Seq analysis included three time points: untreated, TNF-α for 24h, and TNF-α for 24h followed by 48 hr chase. We have added that information in the figure legend. The multiple sampling is actually key to the trajectory analyses shown in subsequent Figures (**New S7 & S8 Figs**).

10. Figure 5: How many replicates were these RNAseq data derived from?
Figure 7C, D: Do data points represent mean and SD of technical replicates from one experiment? Or means from multiple independent experiments?

The RNA-seq experiments included repeats from two independent experiments conducted at 8 months apart.

Data in **Figure7C** and **D** were from triplicates of one ChIP experiment (**New Fig 6C**). We clarified this in the Figure legend.

11. Line 403-404: References for the activity of the described drugs would be useful for reader confirmation

We added the requested new references.

12. Figure 9: enumeration of cell viability with these different combinations of HIV infection and drug treatment are important.

We have provided drug toxicity data in **New S1 Fig.**

13. Figure 9 figure legend C: assume you mean three independent experiments and not technical replicates?

The three repeats of each treatment were conducted on parallel samples in one experiment. They are not three independent experiments conducted at different times.

14. Figure 9D: The un-spliced RNA data is not presented clearly. Assume fold change is calculated from the no treatment wells, yet it is not clear this is 1 given the strange choice in axis (0.9, 1.2?). What are there * above untreated? If this is a comparison to uninfected then this needs to be made clear in the figure legend.

Yes, to calculate the fold changes in the Figure (**New Fig 11**), the mean value of the HIV transcripts from the untreated cells was normalized to “1”. However, there are variations among the different repeats, some of which showed a value lower than “1” while others showed a value slightly higher than “1”. We made changes to the text to provide a clearer explanation.

15. Discussion point: Is Nurr1 expressed/active in macrophage cells? Could a similar mechanism be at work in infected macrophages in the perivascular space?

Nurr1 is expressed in macrophages and many other cells in the brain including neurons and astrocytes. It does seem likely that HIV infection in perivascular macrophages is similarly regulated by Nurr1, however, without any data, we prefer not to speculate about this at this stage.
