# Supplementary material for: Recruitment of the CoREST transcription repressor complexes by Nerve Growth factor IB-like receptor (Nurr1/NR4A2) mediates silencing of HIV in microglial cells
Source: PLoS Pathog. 2022 Jul 7;18(7):e1010110. doi: 10.1371/journal.ppat.1010110 (PMC9295971; doi:10.1371/journal.ppat.1010110)
Supplement: Attachment — Submitted filename: PPATHOGENS-D-21-02310-Reviewer comments.pdf [file ppat.1010110.s013.pdf]

In the manuscript "**The Nerve Growth Factor IB-like Receptor Nurr1 (NR4A2) recruits CoREST transcription repressor complexes to silence HIV following proviral reactivation in microglial cells",** Ye et al and colleagues report on their investigation of the Nerve Growth Factor IB-like nuclear receptor Nurr1 (NR4A2 and its role in restricting HIV transcription in microglia cells. The authors' major finding is that Nurr1 plays an important role in shutting down HIV transcription in microglia cells via the recruitment of the CoREST/HDAC1/G9a/EZH2 transcription repressor complex to the HIV promoter. Using transcriptomic analyses, Ye et al also report that Nurr 1 downregulates several cellular genes associated with inflammation, cell cycle and metabolism to promote HIV silencing and microglia homeostasis.

Major Comments

The authors have made some important observations about the role of Nurr1 in HIV transcription in microglia cells. The manuscript is clearly written and provides partial explanation for the silencing of HIV transcription in microglia following spontaneous reactivation that the authors observed in their previous work. However, there are some limitations to this study. Namely, all the mechanistic work was performed using a transformed microglia cell line. Understandably, accessing brain microglia from ART-treated individuals is impossible, but newly established resources such as the Last Gift Study (Rawlings SA et al, PMID: 34257439) could serve as a post-mortem source of well-preserved microglia cells from ART-infected people to at the very least confirm some of the observations made with agonists and epigenetic drugs. The iPSC derived human microglial cells infected in vitro with a reporter virus while provide some information, still lack the nuances of microglial cells isolated from HIV-infected individuals. Additional points that should be addressed are listed below.

1. The HC69 represents a clonal population of cells with the same integration site. Would the authors conclusion hold true in primary microglia cells isolated from ART-treated individuals where there is likely to be multiple integration site in the microglial cell population?
2. The authors concentrated on Nurr1, however, the western blot analysis in figure 3D would also suggest at least a partial role for Nur77 and Nor1 in HIV silencing.
3. Figure 8A, As these epigenetic drugs are reported to induce HIV expression at least in T cells, an important control to include is the effect of these compounds on cells that have not been treated with TNF-α. Are they simply reactivating silent HIV genomes?
4. Line 161, the authors mentioned that the substantial decrease in GFP+ observed in the chase experiments is unlikely due to GR (glucocorticoid receptor)-mediated HIV silencing as the cells were not cultured in the presence of dexamethasone, a ligand of GR. What were the culture conditions for the cells? This is missing in the methods section. The presence of fetal bovine serum (FBS) could be a potential source of cortisol (GR ligand) to which the cells are exposed to.
5. What is the effect of toxicity of the agonists and epigenetic drugs used on some of the observations? The authors should provide toxicity data on microglial cells.
6. Line 383 and figure 7, there is really no strong evidence to suggest that G9a and HDAC1 decreased at the promoter at 4hr post-TNF-α treatment. In fact, this is clearly not the case in the qPCR results. The minor differences in the peaks in the ChiP seq experiment could be due to experimental fluctuations. Did the authors use a normalization approach, i.e spike in a known quantity of a certain DNA to standardized between samples (e.g., ChIP seq spike in normalization by Active Motif). Also, are the scales the same for the read counts (since they are not shown)? How many times were these experiments performed?
7. In fact in many of the figures , it is not cleared how many times the experiments were repeated (e.g. western blot analyses data). The number of independent experiments performed to demonstrate reproducibility should be clearly stated for all the figures (not just the replicates). Also, the type of statistical tests performed should be indicated in both the figure legends and a statistical analyses plan should be included in the methods.

Minor

1. Line 69 appears to be incomplete: “However, the early studies neglected both the side effects of anti-HIV drugs on neuronal damage which could mask the benefits of reduced HIV expression by cART and the impact of HIV latency on the development of HAND”. Both the side effects of anti HIV drugs and what else?
2. As this is still controversial and under intense investigation, can the authors confirm their statement that microglia are “long-lived” reservoirs in ART-suppressed individuals (line 76). The reference provided (Ref 20) does not provide concrete evidence.
3. In the methods section, instead of randomly listing the antibodies used in the study, to help guide the readers, the antibodies should be listed in accordance with the assay it was used in (e.g. ChiP, immunoblotting, etc.). Are the ChiP antibodies validated for the purpose of chromatin immunoprecipitation for example.
